# Immunogenetic Factors Associated with Severe Respiratory Illness Caused by Zoonotic H1N1 and H5N1 Influenza Viruses

**DOI:** 10.1155/2012/797180

**Published:** 2011-11-03

**Authors:** Jennifer Juno, Keith R. Fowke, Yoav Keynan

**Affiliations:** ^1^Department of Medical Microbiology, University of Manitoba, Winnipeg, MB, Canada R3E 0J9; ^2^Department of Community Health Sciences, University of Manitoba, Winnipeg, MB, Canada R3E 0J9; ^3^Department of Medical Microbiology, University of Nairobi, Nairobi 00100, Kenya; ^4^Department of Internal Medicine, University of Manitoba, Winnipeg, MB, Canada R3E 0J9

## Abstract

Following the 2009 H1N1 pandemic and ongoing sporadic avian-to-human transmission of H5N1 viruses, an emphasis has been placed on better understanding the determinants and pathogenesis of severe influenza infections. Much of the current literature has focused on viral genetics and its impact on host immunity as well as novel risk factors for severe infection (particularly within the H1N1 pandemic). An understanding of the host genetic determinants of susceptibility and severe respiratory illness, however, is currently lacking. By better defining the role of genetic variability in influenza infection and identifying key polymorphisms that impair the host immune response or correlate with protection, we will be able to better identify at-risk populations and new targets for therapeutic interventions and vaccines. This paper will summarize known immunogenetic factors associated with susceptibility or severity of both pH1N1 and H5N1 infections and will also identify genetic pathways and polymorphisms of high relevance for future study.

## 1. Introduction

Transmission of zoonotic influenza A viruses to humans is commonly the cause of new pandemics, which typically result in high disease burden and increased symptomatic severity and mortality. In order to predict which populations may be at highest risk of infection and to develop more effective therapeutic interventions and vaccines, a thorough understanding of both viral and host contribution to pathogenesis is required. In both the recent 2009 H1N1 (pH1N1) pandemic and the on-going rare avian-to-human transmission of H5N1, numerous studies have taken an in-depth look at the impact of viral evolution and mutation on viral pathogenesis. Conversely, while both human and animal model studies of the host immune response to infection have identified correlates of severe disease, the contribution of host genetics to these correlates and to variability in susceptibility remains relatively unknown. Identification of host genetic polymorphisms contributing to altered susceptibility or disease severity has several benefits: identification of high-risk populations at greater need of prophylactic intervention, elucidation of host proteins important in virus-host interactions, and new targets for therapeutic interventions or vaccine development [1]. Studies of host genetics have provided important contributions to the study of other infectious diseases, including HIV, SARS, and HCV. This paper will describe what is currently known about the impact of host immunogenetics in both pH1N1 and H5N1 infections and will identify highly relevant polymorphisms and genetic pathways that could be investigated in future work.

## 2. 2009 Pandemic H1N1

H1N1 influenza viruses emerged as a result of a presumed or documented reassortment of segments from viruses of zoonotic origin with human-adapted influenza virus to cause pandemic spread in 1918 and again in 2009. The 2009 appearance of a swine-origin reassortant virus led to the first pandemic of the 21st century. During earlier pandemics, records indicate that certain individuals or populations appeared to be more susceptible to severe disease, but the ability to conduct studies in order to understand the immune mechanisms that underlay the increased propensity for complications was limited. The 2009 H1N1 (pH1N1) pandemic was accompanied by improved surveillance, thereby facilitating better estimation of disease severity and methods to examine the immune mechanisms behind complicated disease [2–11]. This surveillance allowed for the identification of several novel risk factors among various populations, but with a limited understanding of the genetic variation that may contribute to those risk factors.

### 2.1. Novel Risk Factors Associated with Severity of Pandemic H1N1 Infection

The 1918 H1N1 as well as the recent 2009 pandemics were both notable for the comparatively high rates of morbidity among healthy, young adults not typically observed with seasonal influenza [11]. During the recent pandemic, several studies of confirmed pH1N1 cases in Canada and the US reported the median age of severe infections to be 23–27 years old [8, 10]. In Canada, 30%–48% of infections also presented in persons with comorbidities; diabetes, heart disease, and immunosuppression were associated with the highest risk of severe infection, while lung diseases and obesity were among the most common underlying conditions [10, 12–14]. The role of pregnancy as a risk factor, regardless of the stage, was also supported by a myriad of reports; among hospital admissions, pregnancy accounted for roughly 30% of female cases aged 20–39 years old [9, 12, 15]. 

Ethnicity was another major risk factor of pH1N1 susceptibility identified in several populations in North America and Australasia. The increased proportion of aboriginal individuals presenting with severe pH1N1 infection was not unique for this pandemic and was also seen in the 1918 H1N1 pandemic during which mortality in aboriginal communities in North America (3%–9%) was significantly higher than among nonaboriginal communities [16, 17]. In the 2009 pandemic, Pacific Islanders accounted for 2.5% of the Australian population but made up 9.7% of patients admitted to Australian ICUs with confirmed pH1N1. Maori individuals represent 13.6% of the New Zealand population, but accounted for 25% of ICU admissions in the ANZIC study [18]. Kumar et al. [12] also reported 25.6% of the individuals admitted to ICUs in Canada belonged to First Nations, Inuit, Metis, or aboriginal ethnicities; this is an overrepresentation compared to the 4.4% rate of self-reported aboriginal ethnicity according to the 2001 census (Statistics Canada). Similarly, pH1N1 mortality rates among American-Indian/Alaska Natives were four times higher than persons in all other ethnic populations combined in the United States [19].

None of these studies examined the causal factors that lead to the higher influenza mortality in the high-risk groups described. It is clear that multiple converging risks account for the high rates of complications, including socioeconomic factors such as inability to access care, delayed seeking of care, higher rates of poverty, and greater numbers of household members. A few of the risk factors listed in the previous sections, however, share a degree of immune system impairment. One can, therefore, speculate that the partial protection afforded by the immune system, primarily by cross-reactive CD8+ T-cells recognizing viral epitopes, is decreased in some of the previously described groups (pregnancy is a good example). Additionally, genetic variation in immune-related genes leading to either gain-of-function or loss-of-function phenotypes could contribute to the variation observed in pH1N1 susceptibility and disease severity.

### 2.2. Novel Immunogenetic Risk Factors Associated with Severity of Pandemic H1N1 Infection

When a novel strain of influenza emerges, the pre-existing antibody response directed largely at the surface glycoproteins is rendered ineffective. In these cases, the mechanisms underlying heterosubtypic cross-protection assume a dominant role and it is, therefore, not surprising that immune dysfunction caused by underlying genetic polymorphisms may lead to impaired responses and would, therefore, be associated with adverse outcomes. During the 2009 H1N1 pandemic, several immunogenetic determinants of severe disease were identified.

When a novel strain of influenza emerges, the pre-existing antibody response directed largely at the surface glycoproteins is rendered ineffective. In these cases, the mechanisms underlying heterosubtypic cross-protection assume a dominant role and it is, therefore, not surprising that immune dysfunction caused by underlying genetic polymorphisms may lead to impaired responses and would therefore be associated with adverse outcomes. During the 2009 H1N1 pandemic, several immunogenetic determinants of severe disease were identified.

#### 2.2.1. CCR5Δ32 Allele

The CCR5 protein is a chemokine receptor expressed primarily on T cells, macrophages, and dendritic cells. CCR5 plays a pivotal role in mediating leukocyte chemotaxis in response to chemokines (including RANTES, MIP-1*α*, and MIP-1*β*) and is believed to be important in the homing of many immune cell subsets, including regulatory T cells and Th17 cells, to mucosal surfaces. Until recently, the purported role of CCR5 in supporting the antiviral immune response was limited to appreciation of the effect of receptor deficiency in protecting from HIV infection and disease progression among individuals homozygous for the Δ32 allele. The understanding of the roles played by CCR5 was expanded when the Δ32 allele was found to be associated with an increased risk of symptomatic and fatal West Nile Virus (WNV) infection [20–22], a severe adverse reaction to the live yellow fever virus vaccine, and with severe tick-borne encephalitis symptoms [23, 24]. Together, these data suggest that CCR5 may also play a critical role in the immune response to flavivirus infections. The spectrum of symptomatic severity observed during the 2009 H1N1 pandemic led our group to study CCR5 genotype among patients requiring intensive care admission and respiratory support for severe H1N1 symptoms. Among twenty samples of confirmed severe pH1N1 infection, the CCR5Δ32 allele was found in 5 out of 9 of the Caucasian individuals, giving a Caucasian allele frequency of 27.8% [25] (Table 1). This observed frequency is approximately 2.5 times higher than that reported for local North American Caucasian populations [26, 27]. Given the small sample size available in this cohort, further studies will be required to conclusively determine the impact of CCR5 deficiency on pH1N1 susceptibility and severity.

#### 2.2.2. IgG2 Subclass Deficiency

Similarly to IgG1, IgG2a and IgG2b are able to bind to Fc receptors with high affinity and are thought to be important in protecting against influenza infection. A group of Australian investigators identified an index case of severe influenza in a pregnant woman with IgG2 subclass deficiency and subsequently measured total IgG and IgG subclasses in all patients with pH1N1 infection requiring ICU care (many of whom were pregnant) compared to less severe controls and asymptomatic pregnant women presenting to antenatal clinic. A low level of IgG2 was correlated with severe pH1N1 infection after multivariate analysis. Measurement of IgG2 after 90 days among 15 of the surviving IgG2-deficient patients showed that 11 remained IgG2 deficient despite albumin levels returning to baseline values [28]. Additionally, a case-control study from China enrolled 38 Asian patients with respiratory failure due to severe pandemic influenza and compared IgG2 levels with 36 mild cases. They did not find any cases of selective IgG2 deficiency, but did observe significantly lower levels of IgG2 among the severe cases (despite normal levels of the other IgG subclasses) [29]. The authors looked for the presence of *Fc*γ*RIIa* and *IGHG2* genotypes (Table 1) that were previously shown to be associated with IgG2 deficiency, but found similar rates among cases and controls. They did, however, corroborate the previously reported finding [30] of cytokine dysregulation among severe cases of infection and suggested that the mechanism responsible for the low IgG2 is the more robust Th1 response and a suppressed Th2 response. Given the lack of *Fc*γ*RIIa* and *IGHG2* genotype data available from the Gordon et al. study [28], the impact of these polymorphisms on IgG2 levels and severe pH1N1 infection remains to be determined.

### 2.3. Additional Candidate Polymorphisms Contributing to Severe Influenza

Genetic polymorphisms associated with pH1N1 susceptibility and disease severity identified to date are limited, and much of the data is derived from small cohorts. An improved understanding of the sequence of immune responses to influenza as well as the application of newer technologies that employ high-throughput expression array or sequencing technologies can be used to guide a more focused approach to identify specific pathways that may be differentially activated by individuals with severe disease. Based on available data, we can identify several immune pathways and their genetic variants that warrant further investigation.

#### 2.3.1. NLRP Inflammasomes

Recently, emphasis has been given to the role of the inflammasome in viral infections and, specifically, influenza. NLRP3 (NOD-like receptor family, pyrin domain-containing 3) inflammasomes are multiprotein complexes containing NLRP3, ASC (or Pycard), and caspase 1. Activation of cytokine/chemokine release through NLRP3 requires a signal derived from Toll-like receptor (TLR) stimulation, with resultant production of pro-IL-1*β*, -IL-18, and -IL-33. The prointerleukins are in turn cleaved to their respective active forms by caspase 1, which requires the input of an additional signal. The second signal in the context of influenza has been elegantly demonstrated by Ichinohe et al. [31], who showed that golgi-localized H1N1 M2 is both necessary and sufficient to trigger inflammasome activation. Differential expression of the components of the two signaling cascades that are required for inflammasome activation may therefore explain differences in influenza disease severity. Indeed, mouse knockout studies have shown that intact inflammasomes are necessary for innate immune responses to influenza A, chemokine production, and late stage viral clearance (reviewed in [32]). Evidence suggests that NLRP3-mediated signaling is also important in cellular recruitment and tissue repair during infection. A similar requirement for proper ASC function was observed in adaptive influenza immune responses. Multiple *NLRP3* SNPs have been associated with dysregulated inflammation responses and NLRP3 mRNA stability in humans but have not been examined in the context of pH1N1 susceptibility or mortality [33, 34] (Table 1).

#### 2.3.2. HLA Alleles

The CD8+ T-cell response is a strong predictor of vaccine-induced protection and is thought to be particularly valuable in the elderly. Because this response is focused on more conserved viral proteins, it has the additional benefit of providing some cross-reaction with new influenza strains [46, 47]. Undoubtedly, multiple factors underlie the differences in disease severity among ethnic groups, as previously discussed. From an immunogenetic perspective, however, HLA alleles are among the most variable human genes, and it is therefore conceivable that variable proportions of HLA class I alleles among ethnic groups may lead to qualitatively and quantitatively distinct CD8+ T-cell responses, as well as differences in immunodominant epitopes (Table 1). Boon et al. [35] have demonstrated that the frequency of CTL responses specific for the HLA-B8-restricted epitope NP_380–388_ was lower in HLA-B27-positive donors than in HLA-B27-negative donors. They also showed that the HLA-A1-restricted epitope NP_44–52_ responses were higher in HLA-A1-, -A2-, -B8-, and -B35-positive donors than in other donors. These observations suggest that the epitope specificity and magnitude of the CTL response is related to the HLA class I genetic background [35]. 

#### 2.3.3. Gene Expression Studies

The role of cell-mediated immunity in ameliorating infection caused by novel influenza strains has been the focus of intense study [48] and it is, therefore, the most compelling area to investigate in order to identify immunogenetic factors that predict severity of pandemic H1N1 influenza. A comprehensive investigation was undertaken by Bermejo-Martin et al. [49] in a study from Spain. They enrolled 19 critically ill patients with primary pH1N1 influenza pneumonia and used gene expression analysis in order to identify host immune responses associated with severe disease defined by illness requiring mechanical ventilation. They identified impaired expression of a number of MHC class II and MHC class I genes, T-cell receptor-associated genes, and also of a cluster of genes thought to be involved in dendritic cell maturation, indicating defective antigen presentation in the most severe group of patients. They found further evidence for the effect of altered antigen presentation on the development of an appropriate adaptive response against the virus in the impaired expression of a group of genes critical to the activation and function of both T and B cells. The group with severe illness also showed higher expression of genes involved in IL-6 and IL-10 pathways, and these results were in concordance with the high serum levels of IL-6 and IL-10 in the group dependant on mechanical ventilation. The authors concluded that severe disease is associated with an impaired transition from innate to adaptive immunity in response to the pH1N1 virus, similar to observations in the context of SARS and severe infections caused by H5N1. The impaired adaptive response was also associated with delayed viral clearance. This study did not, however, explore the role of genetic polymorphisms in this immune dysregulation.

## 3. H5N1 Avian Influenza

Pathogenic avian influenza A/H5N1 viruses are endemic among poultry populations across Asia and Africa and present an ongoing risk for avian-to-human transmission. As of June 22, 2011, the WHO reports a total of 562 confirmed H5N1 cases and 329 deaths across 15 countries worldwide [50]. Although human-to-human transmission of H5N1 has so far been rare, the potential for viral evolution into a more transmissible strain raises the possibility that H5N1viruses could cause a pandemic. Given the high mortality rate currently associated with human H5N1 infection, a thorough understanding of the immune response and the underlying mechanisms of viral pathogenesis is crucial to improve treatment and to identify highly susceptible populations. To date, much of the research into the immunobiology and pathogenesis of human H5N1 infection has focused on the H5 haemagglutinin protein and the impact of viral genetic polymorphisms on viral pathogenicity. Although studies have begun to characterize of the role of the host immune response in pathogenesis, the impact of genetic variability on susceptibility and disease severity remains an important gap in our current knowledge. By identifying host genetic polymorphisms that exacerbate immunopathology or provide protection, we will be able to improve treatment and future vaccines [1, 51].

### 3.1. The Case for Host Genetic Variation in H5N1 Susceptibility and Disease Severity

The precise impact of host genetic variability on H5N1 susceptibility remains somewhat controversial, given the limited case data available and the relatively low number of published studies. A case study in Indonesia [52] found evidence of clusters of H5N1 infection among blood relatives that may be indicative of shared genetic susceptibility, but the authors were unable to rule out a shared viral exposure or altered viral pathogenesis in any of the clusters. Similar observations in a number of additional studies have prompted several authors to suggest a potentially strong genetic basis for H5N1 susceptibility [53–57]. A compilation of confirmed H5N1 cases worldwide found that, on average, 22% of cases occurred in clusters, and only 6% of cases within the clusters were not genetically related to other cluster members [1]. While this data does not conclusively point to genetic variation as an important determinant of susceptibility, it is important to note that human-to-human transmission of H5N1 is very rare, and therefore does not likely explain the high degree of genetic relatedness among cases [1]. It would also be expected that clusters of nonrelated individuals working in the poultry industry would be more prominent than genetically related clusters, if people are at equal risk of infection [1]. Some studies, however, have suggested that the observed clustering of infections among families could occur due to chance alone at the low rates of infection that are observed in H5N1 and highlight the difficulty in drawing conclusions from the currently available data [58]. 

### 3.2. Candidate Genes Influencing Susceptibility

Despite the intense research into the effect of viral mutations on pathogenesis and viral fitness, very few, if any, studies have assessed the impact of specific host polymorphisms on human H5N1 infection. Although genetic association studies face a number of challenges with regard to H5N1 infection, including limited numbers of infected patients, difficulty in determining appropriate control populations, and limited human-to-human transmission with resultant high likelihood of exposure to unique virus, identifying genetic variants involved in increased susceptibility and/or disease outcome could provide important data regarding crucial host/virus interactions and the qualities of a protective immune response. To date, a number of candidate genes have been identified from both human and mouse immunobiology studies. Mouse models of influenza infection have the advantage of being able to dissect gene expression kinetics and the characteristics of the immune response at various stages of infection. Comparison of infection across inbred mouse lines demonstrates significant differences in viral titre and core temperature, as well as distinct patterns of immune gene upregulation, suggesting an important contribution of host genetic background [59].

#### 3.2.1. Host-Virus Interactions

Genetic variation affecting host proteins required for viral entry and pathogenesis may partially explain the sporadic and rare nature of avian-to-human H5N1 transmission. Although humans do express the SA*α*-2,3Gal molecules that are efficiently bound by avian H5N1, their expression is usually limited to the lower respiratory tract and has only occasionally been detected in the nasal mucosa and upper respiratory tract [60, 61]. Additionally, the binding of several influenza strains to human erythrocytes is highly variable, with up to a 40-fold difference between individuals tested in one study, suggesting a role for genetic polymorphism in regulating susceptibility [62]. Whether variation in the human *ST3 beta-galactosamide alpha-2,3-sialyltranferase 1* (*ST3GAL1*) gene that produces the SA*α*-2,3Gal linkage affects H5N1 susceptibility is not known, but remains a possibility [63]. Interestingly, a population genetics study designed to detect human SNPs under virus-driven selective pressure found a significant enrichment of glycan biosynthesis gene SNPs associated with viral selection, including rs3758105 (intronic A/G SNP) in the *ST3GAL1* gene [64] (Table 2). Data demonstrating the infection of upper respiratory tract cells with H5N1 *in vitro* also suggests the presence of additional cellular receptors for the virus [61]. 

Alternately, prevention of viral attachment in the respiratory tract is accomplished by host proteins that can sterically hinder viral HA binding, or aggregate and opsonize the virus. These proteins include serum mannose-binding lectin 2 (MBL2) and surfactant, pulmonary-associated protein A1 and D (SFTPA1 and SFTPD, resp.). A SNP in *MBL2* (230G/A), resulting in low serum MBL levels, is associated with SARS susceptibility [36, 37], while polymorphisms in *SFTPA1* and *SFTPD* are associated with other respiratory illnesses [65, 66] (Table 2). To date, none of these polymorphisms have been investigated with respect to H5N1 susceptibility.

#### 3.2.2. Innate Immune Signalling

Induction of an innate immune response following infection can occur as a result of the activation of pattern recognition receptors (PRRs), commonly known as Toll-like receptors (TLRs). TLRs recognize many elements of foreign pathogens, including LPS, flagellin, and dsRNA, and initiate signaling cascades that result in the production of type I interferons. Accumulating data suggests that genetic variation in TLRs and their associated signaling components modulates the response to TLR ligands and, consequently, the inflammatory immune response [67]. TLR3 is constitutively expressed on lung alveolar and bronchial epithelial cells and has been shown to contribute to the secretion of multiple cytokines following influenza A infection [68]. Given the data suggesting that H5N1 pathogenicity is due in part to alterations in innate immune responses and hypercytokinemia, it is plausible that polymorphisms altering TLR function could contribute to susceptibility or protection from infection. This hypothesis is supported by a genetic study of a case of influenza-associated encephalopathy, a condition associated with apoptosis and hypercytokinemia [45]. In this case, a missense mutation (908T/C) in the TLR3 gene was identified and was shown to be a loss-of-function mutation, suggesting a protective role for TLR3 signaling in severe influenza infection [45] (Table 2). Although these results are consistent with studies suggesting a protective effect of TLR3 in West Nile infection [69], they are at odds with TLR3 null mouse studies, which have shown reduced proinflammatory cytokine production following cellular stimulation [70–72]. Consequently, the contribution of TLR genetic variants to H5N1 inflammatory responses remains to be resolved.

#### 3.2.3. Interferon-Related Pathways

Induction of the type I interferon response during influenza infection appears to be important in both human and mouse models, as evidenced by the increased expression of genes including *Irf1, Ifi202*, *Oas1*, and *Mx1* in mouse microarray studies [73–75] (reviewed in [76]). This is consistent with the observation that viral evasion and attenuation of the IFN pathway contributes to H5N1 pathogenesis in humans and suggests potential targets for genetic studies [75, 77]. A strong target for analysis includes the myxovirus resistance (*Mx*) gene, which encodes interferon-induced antiviral proteins that inhibit viral RNA transcription and consequently confer influenza resistance in mouse lines with functional *Mx1* alleles [78]. Polymorphisms in swine *Mx* genes have also been associated with influenza susceptibility [79] and multiple SNPs in the human *MxA* gene have been associated with variability in IFN responsiveness in Hepatitis C infection [80] and SARS susceptibility [38–40] (Table 2). Specifically, the −123C/A promoter SNP associated with SARS protection correlates with increased basal MxA expression, leading the authors to speculate that −123 genotype may be an important determinant of H5N1 susceptibility [39]. Although human MxA protein has been shown to inhibit influenza replication [81], no studies have looked for an association between *MxA* SNPs and H5N1 disease outcome or susceptibility. Because *MxA* is located on chromosome 21, studies have compared susceptibility to respiratory infections between wild-type and trisomy 21 patients (who exhibit increased MxA expression), but found greater susceptibility among the trisomy 21 group [82]. This group of patients is known to suffer from a multilevel T-cell dysfunction, however, making the role of MxA in respiratory immunity somewhat unclear. 

Polymorphisms in *OAS1* (2′,5′-oligoadenylate synthetase 1; an interferon-induced antiviral protein) have also been associated with SARS susceptibility and progression [38, 40] and West Nile infection [41]. Consistent with the idea of increased H5N1 susceptibility and pathogenesis associated with poor IFN responses, the *OAS1* SNP rs1077467 is correlated with reduced OAS-1 protein activity and associated with increased susceptibility to West Nile infection and *in vitro* viral replication [41]. 

#### 3.2.4. Cytokine Response

Comparison of severe H5N1 infections with uncomplicated seasonal influenza infections revealed a pattern of increased viral load and elevated cytokine production in the respiratory tract and serum [75, 83]. The robust cytokine/chemokine response often seen in H5N1 infected patients (hypercytokinemia) is believed to be at least partially responsible for the observed pathogenesis and high fatality of H5N1 infection. Elevated cytokines both *in vivo *and *in vitro* include IFN*γ*, sIL-2R, IL-6, IP-10, TNF*α*, and MCP-1 [75, 84–86]. Mouse models of H5N1 infection also demonstrate elevated levels of MCP-1, MIP-1*α*, IL-6, and IFN*γ*, even compared to 1918 H1N1 virus infection [87]. Knocking out IL-1R in mice exacerbates H5N1 pathology and suggests that IL-1*β*-mediated signalling may be important in protection [75]. In ferret models, IP-10 upregulation and signaling through CXCR3 was determined to be a major component of H5N1 disease severity and mortality [88]. Expression of many of these chemokines and cytokines in humans is modulated by SNPs in their promoter regions, including MCP-1 −2518 G/A [89], IP-10 −201G/A [90], and IL-6 −174G/C [91]. Genetic variants affecting expression and function of chemokine receptors may also modulate influenza pathogenesis, as CCR5 knock-out mice exhibit increased influenza mortality, whereas CCR2 knockout strains show increased survival (Table 2); both of these effects appear to be related to the kinetics and strength of macrophage recruitment to the lung [42]. *In vitro* evidence further suggests upregulation of CCR5 on monocyte-derived macrophages that may enhance pathogenesis [92].

## 4. Conclusions

Although relatively few studies have systematically evaluated the influence of genetic polymorphisms on susceptibility and disease severity in zoonotic H1N1 and H5N1 infections, the data available suggest that host immunogenetic variation could play an important role in determining the outcome of the immune response. With improvements in surveillance and case confirmation as well as new sequencing and gene expression platforms, we now have the capability to study host genetic variants among severe respiratory illness cases. Although several challenges to conducting such a study include ethical permission to carry out genetic polymorphism studies, the need for large numbers of well-characterised clinical specimens with relevant clinical data, difficulty to obtain sufficient number of samples from severe and fatal cases at a single institution, and difficulty in identifying mild controls. The extreme cases of human H5N1 disease are very rare, sporadic, with scattered cases in different countries, adding economic and political sensitivities associated with this disease. Overcoming these barriers and conducting collaborative research can lead to insights that will shed light on the varying degree of susceptibility observed between populations during the recent H1N1 pandemic and will provide greater insight into the host-pathogen interactions that determine disease course during severe H5N1 infection. 

## Figures and Tables

**Table 1:** Genetic polymorphisms of interest in H1N1 susceptibility and severity.

Gene	Polymorphism	Functional significance	Reference
CCR5	CCR5∆32	Increased allele frequency among Canadian H1N1 ICU cases	[25]
Fc*γ*RIIa, IGHG2	IGHG2 *n/*-n Fc*γ*RIIa-R131H	Polymorphisms previously linked to IgG2 deficiency, but not corroborated in H1N1 patients	[28–30]
NLRP3	2107C/A (Q705 K) rs4612666 (intron 7) rs10754558 (3′ UTR)	Association with dysregulation of inflammatory response (2107), alteration of NLRP3 mRNA stability and enhancer activity	[33, 34]
HLA	Various alleles	Influenza-specific CTL responses exhibit varying frequency and magnitude across various HLA alleles	[35]

**Table 2:** Genetic polymorphisms of interest in H5N1 susceptibility and severity.

Gene	Polymorphism	Functional significance	References
MBL2	230G/A	Low serum MBL levels; increased susceptibility to SARS	[36, 37]
MxA	−88G/T (rs2071430) −123C/A (rs17000900)	Increased basal (−123 A) and IFN-stimulated (−88 T) MxA expression and activity *in vitro;* altered susceptibility to SARS	[38, 39]
OAS1	rs2660 (3′ UTR A/G) rs3741981 (Exon 3 A/G) rs1077467 (Intron 5)	Altered susceptibility to SARS (3′ UTR, Exon 3); West Nile susceptibility and reduced activity due to splicing (Intron 5)	[38, 40, 41]
CCR5	CCR5∆32	Increased mortality among CCR5 knockout mice, increased allele frequency among severe H1N1 infections	[25, 42]
CCR2	190G/A (V64I)	Altered HIV progression; stabilization of CCR2a splice variant and binding to CCR5	[43, 44]
TLR3	908T/C	Missense mutation identified in a patient with influenza-associated encephalopathy	[45]

## References

[1] Horby P, Sudoyo H, Viprakasit V (2010). What is the evidence of a role for host genetics in susceptibility to influenza A/H5N1?. *Epidemiology and Infection*.

[2] Presanis AM, de Angelis D, Hagy A (2009). The severity of pandemic H1N1 influenza in the United States, from April to July 2009: a Bayesian analysis. *PLoS Medicine*.

[3] Presanis AM, Lipsitch M, Daniela DA (2009). The severity of pandemic H1N1 influenza in the United States, April–July 2009. *PLoS Currents*.

[4] Archer B, Cohen C, Naidoo D (2009). Interim report on pandemic H1N1 influenza virus infections in South Africa, April to October 2009: epidemiology and factors associated with fatal cases. *Euro Surveillance*.

[5] Donaldson LJ, Rutter PD, Ellis BM (2009). Mortality from pandemic A/H1N1 2009 influenza in England: public health surveillance study. *BMJ*.

[6] European Centre for Disease Prevention and Control (ECDC) (2011). Announced cumulative number of confirmed fatal cases of 2009 pandemic influenza A(H1N1).

[7] Echevarría-Zuno S, Mejía-Aranguré JM, Mar-Obeso AJ (2009). Infection and death from influenza A H1N1 virus in Mexico: a retrospective analysis. *The Lancet*.

[8] Louie JK, Acosta M, Winter K (2009). Factors associated with death or hospitalization due to pandemic 2009 influenza A(H1N1) infection in California. *Journal of the American Medical Association*.

[9] Vaillant L, la Ruche G, Tarantola A, Barboza P (2009). Epidemiology of fatal cases associated with pandemic H1N1 influenza 2009. *Euro Surveillance*.

[10] Campbell A, Rodin R, Kropp R (2010). Risk of severe outcomes among patients admitted to hospital with pandemic (H1N1) influenza. *CMAJ*.

[11] Girard MP, Tam JS, Assossou OM, Kieny MP (2010). The 2009 A (H1N1) influenza virus pandemic: a review. *Vaccine*.

[12] Kumar A, Zarychanski R, Pinto R (2009). Critically ill patients with 2009 influenza A(H1N1) infection in Canada. *Journal of the American Medical Association*.

[13] Zarychanski R, Stuart TL, Kumar A (2010). Correlates of severe disease in patients with 2009 pandemic influenza (H1N1) virus infection. *CMAJ*.

[14] Hanslik T, Boelle PY, Flahault A (2010). Preliminary estimation of risk factors for admission to intensive care units and for death in patients infected with A(H1N1)2009 influenza virus, France, 2009-2010. *PLoS Currents*.

[15] Creanga AA, Johnson TF, Graitcer SB (2010). Severity of 2009 pandemic influenza A (H1N1) virus infection in pregnant women. *Obstetrics and Gynecology*.

[16] Graham-Cumming G (1967). Health of the original Canadians, 1867–1967. *Medical Services Journal*.

[17] Johnson NP, Mueller J (2002). Updating the accounts: global mortality of the 1918–1920 “Spanish” influenza pandemic. *Bulletin of the History of Medicine*.

[18] Webb SAR, Pettilä V, Seppelt I (2009). Critical care services and 2009 H1N1 influenza in Australia and New Zealand. *The New England Journal of Medicine*.

[19] Centers for Disease Control and Prevention (CDC) (2009). Deaths related to 2009 pandemic influenza A (H1N1) among American Indian/Alaska Natives—12 states, 2009. *Morbidity and Mortality Weekly Report*.

[20] Glass WG, Lim JK, Cholera R, Pletnev AG, Gao JL, Murphy PM (2005). Chemokine receptor CCR5 promotes leukocyte trafficking to the brain and survival in West Nile virus infection. *Journal of Experimental Medicine*.

[21] Glass WG, McDermott DH, Lim JK (2006). CCR5 deficiency increases risk of symptomatic West Nile virus infection. *Journal of Experimental Medicine*.

[22] Lim JK, Louie CY, Glaser C (2008). Genetic deficiency of chemokine receptor CCR5 is a strong risk factor for symptomatic West Nile virus infection: a meta-analysis of 4 cohorts in the US epidemic. *Journal of Infectious Diseases*.

[23] Pulendran B, Miller J, Querec TD (2008). Case of yellow fever vaccine-associated viscerotropic disease with prolonged viremia, robust adaptive immune responses, and polymorphisms in CCR5 and RANTES genes. *Journal of Infectious Diseases*.

[24] Kindberg E, Mickiene A, Ax C (2008). A deletion in the chemokine receptor 5 (CCR5) gene is associated with tickborne encephalitis. *Journal of Infectious Diseases*.

[25] Keynan Y, Juno J, Meyers A (2010). Chemokine receptor 5 big up tri, open32 allele in patients with severe pandemic (H1N1) 2009. *Emerging Infectious Diseases*.

[26] Singh KK, Barroga CF, Hughes MD (2004). Prevalence of chemokine and chemokine receptor polymorphisms in seroprevalent children with symptomatic HIV-1 infection in the United States. *Journal of Acquired Immune Deficiency Syndromes*.

[27] Downer MV, Hodge T, Smith DK (2002). Regional variation in CCR5-Δ32 gene distribution among women from the US HIV Epidemiology Research Study (HERS). *Genes and Immunity*.

[28] Gordon CL, Johnson PDR, Permezel M (2010). Association between severe pandemic 2009 influenza A (H1N1) virus infection and immunoglobulin G(2) subclass deficiency. *Clinical Infectious Diseases*.

[29] Chan JF-W, To KK-W, Tse H (2011). The lower serum immunoglobulin G2 level in severe cases than in mild cases of pandemic H1N1 2009 influenza is associated with cytokine dysregulation. *Clinical and Vaccine Immunology*.

[30] Bermejo-Martin JF, de Lejarazu RO, Pumarola T (2009). Th1 and Th17 hypercytokinemia as early host response signature in severe pandemic influenza. *Critical Care*.

[31] Ichinohe T, Pang IK, Iwasaki A (2010). Influenza virus activates inflammasomes via its intracellular M2 ion channel. *Nature Immunology*.

[32] Pang IK, Iwasaki A (2011). Inflammasomes as mediators of immunity against influenza virus. *Trends in Immunology*.

[33] Verma D, Lerm M, Julinder RB, Eriksson P, Söderkvist P, Särndahl E (2008). Gene polymorphisms in the NALP3 inflammasome are associated with interleukin-1 production and severe inflammation relation to common inflammatory diseases?. *Arthritis and Rheumatism*.

[34] Hitomi Y, Ebisawa M, Tomikawa M (2009). Associations of functional NLRP3 polymorphisms with susceptibility to food-induced anaphylaxis and aspirin-induced asthma. *Journal of Allergy and Clinical Immunology*.

[35] Boon ACM, de Mutsert G, Graus YMF (2002). The magnitude and specificity of influenza A virus-specific cytotoxic T-lymphocyte responses in humans is related to HLA-A and—B phenotype. *Journal of Virology*.

[36] Ip WKE, Kwok HC, Law HKW (2005). Mannose-binding lectin in severe acute respiratory syndrome coronavirus infection. *Journal of Infectious Diseases*.

[37] Zhang H, Zhou G, Zhi L (2005). Association between mannose-binding lectin gene polymorphisms and susceptibility to severe acute respiratory syndrome coronavirus infection. *Journal of Infectious Diseases*.

[38] He J, Feng D, de Vlas SJ (2006). Association of SARS susceptibility with single nucleic acid polymorphisms of OASI and MxA genes: a case-control study. *BMC Infectious Diseases*.

[39] Ching JCY, Chan KYK, Lee EHL (2010). Significance of the Myxovirus resistance A (MxA) gene—123C>a single-nucleotide polymorphism in suppressed interferon *β* induction of severe acute respiratory syndrome coronavirus infection. *Journal of Infectious Diseases*.

[40] Hamano E, Hijikata M, Itoyama S (2005). Polymorphisms of interferon-inducible genes OAS-1 and MxA associated with SARS in the Vietnamese population. *Biochemical and Biophysical Research Communications*.

[41] Lim JK, Lisco A, McDermott DH (2009). Genetic variation in OAS1 is a risk factor for initial infection with West Nile virus in man. *PLoS Pathogens*.

[42] Dawson TC, Beck MA, Kuziel WA, Henderson F, Maeda N (2000). Contrasting effects of CCR5 and CCR2 deficiency in the pulmonary inflammatory response to influenza A virus. *American Journal of Pathology*.

[43] Lee B, Doranz BJ, Rana S (1998). Influence of the CCR2-V64I polymorphism on human immunodeficiency virus type 1 coreceptor activity and on chemokine receptor function of CCR2b, CCR3, CCR5, and CXCR4. *Journal of Virology*.

[44] Nakayama EE, Tanaka Y, Nagai Y, Iwamoto A, Shioda T (2004). A CCR2-V64I polymorphism affects stability of CCR2A isoform. *AIDS*.

[45] Hidaka F, Matsuo S, Muta T, Takeshige K, Mizukami T, Nunoi H (2006). A missense mutation of the Toll-like receptor 3 gene in a patient with influenza-associated encephalopathy. *Clinical Immunology*.

[46] Powers DC, Manning MC, Hanscome PJ, Pietrobon PJF (1995). Cytotoxic T lymphocyte responses to a liposome-adjuvanted influenza A virus vaccine in the elderly. *Journal of Infectious Diseases*.

[47] McElhaney JE, Xie D, Hager WD (2006). T cell responses are better correlates of vaccine protection in the elderly. *Journal of Immunology*.

[48] Thomas PG, Keating R, Hulse-Post DJ, Doherty PC (2006). Cell-mediated protection in influenza infection. *Emerging Infectious Diseases*.

[49] Bermejo-Martin JF, Martin-Loeches I, Rello J (2010). Host adaptive immunity deficiency in severe pandemic influenza. *Critical Care*.

[50] World Health Organization (2011). Cumulative number of confirmed human cases of avian influenza A/(H5N1). *Reported to WHO*.

[51] Mubareka S, Palese P (2008). Human genes and influenza. *Journal of Infectious Diseases*.

[52] Kandun IN, Tresnaningsih E, Purba WH (2008). Factors associated with case fatality of human H5N1 virus infections in Indonesia: a case series. *The Lancet*.

[53] Olsen SJ, Ungchusak K, Sovann L (2005). Family clustering off avian influenza A (H5N1). *Emerging Infectious Diseases*.

[54] Beigel JH, Farrar J, Han AM (2005). Avian influenza A (H5N1) infection in humans. *The New England Journal of Medicine*.

[55] Van Kerkhove MD, Ly S, Holl D (2008). Frequency and patterns of contact with domestic poultry and potential risk of H5N1 transmission to humans living in rural Cambodia. *Influenza and other Respiratory Viruses*.

[56] Hien NT, Farrar J, Horby P (2008). Person-to-person transmission of influenza A (H5N1). *The Lancet*.

[57] Sedyaningsih ER, Isfandari S, Setiawaty V (2007). Epidemiology of cases of H5N1 virus infection in Indonesia, July 2005–June 2006. *Journal of Infectious Diseases*.

[58] Pitzer VE, Olsen SJ, Bergstrom CT, Dowell SF, Lipsitch M (2007). Little evidence for genetic susceptibility to influenza A (H5N1) from family clustering data. *Emerging Infectious Diseases*.

[59] Toth LA, Verhulst SJ (2003). Strain differences in sleep patterns of healthy and influenza-infected inbred mice. *Behavior Genetics*.

[60] Shinya K, Ebina M, Yamada S, Ono M, Kasai N, Kawaoka Y (2006). Avian flu: nfluenza virus receptors in the human airway. *Nature*.

[61] Nicholls JM, Chan MCW, Chan WY (2007). Tropism of avian influenza A (H5N1) in the upper and lower respiratory tract. *Nature Medicine*.

[62] Rumyantsev SN (2006). Genetic immunity and influenza pandemics. *FEMS Immunology and Medical Microbiology*.

[63] Zhang L, Katz JM, Gwinn M, Dowling NF, Khoury MJ (2009). Systems-based candidate genes for human response to influenza infection. *Infection, Genetics and Evolution*.

[64] Fumagalli M, Pozzoli U, Cagliani R (2010). Genome-wide identification of susceptibility alleles for viral infections through a population genetics approach. *PLoS Genetics*.

[65] Thomas NJ, Fan R, DiAngelo S, Hess JC, Floros J (2007). Haplotypes of the surfactant protein genes A and D as susceptibility factors for the development of respiratory distress syndrome. *Acta Paediatrica*.

[66] Lahti M, Löfgren J, Marttila R (2002). Surfactant protein D gene polymorphism associated with severe respiratory syncytial virus infection. *Pediatric Research*.

[67] Schröder NWJ, Schumann RR (2005). Single nucleotide polymorphisms of Toll-like receptors and susceptibility to infectious disease. *The Lancet Infectious Diseases*.

[68] Guillot L, le Goffic R, Bloch S (2005). Involvement of Toll-like receptor 3 in the immune response of lung epithelial cells to double-stranded RNA and influenza A virus. *Journal of Biological Chemistry*.

[69] Daffis S, Samuel MA, Suthar MS, Gale M, Diamond MS (2008). Toll-like receptor 3 has a protective role against West Nile virus infection. *Journal of Virology*.

[70] Alexopoulou L, Holt AC, Medzhitov R, Flavell RA (2001). Recognition of double-stranded RNA and activation of NF-*κ*B by Toll-like receptor 3. *Nature*.

[71] Majde JA, Kapás L, Bohnet SG, De A, Krueger JM (2010). Attenuation of the influenza virus sickness behavior in mice deficient in Toll-like receptor 3. *Brain, Behavior, and Immunity*.

[72] le Goffic R, Balloy V, Lagranderie M (2006). Detrimental contribution of the Toll-like receptor (TLR)3 to influenza A virus-induced acute pneumonia. *PLoS Pathogens*.

[73] Ding M, Lu L, Toth LA (2008). Gene expression in lung and basal forebrain during influenza infection in mice. *Genes, Brain and Behavior*.

[74] Haller O, Staeheli P, Kochs G (2007). Interferon-induced Mx proteins in antiviral host defense. *Biochimie*.

[75] Maines TR, Szretter KJ, Perrone L (2008). Pathogenesis of emerging avian influenza viruses in mammals and the host innate immune response. *Immunological Reviews*.

[76] Trammell RA, Toth LA (2008). Genetic susceptibility and resistance to influenza infection and disease in humans and mice. *Expert Review of Molecular Diagnostics*.

[77] Zeng H, Goldsmith C, Thawatsupha P (2007). Highly pathogenic avian influenza H5N1 viruses elicit an attenuated type I interferon response in polarized human bronchial epithelial cells. *Journal of Virology*.

[78] Vanlaere I, Vanderrijst A, Guénet JL, de Filette M, Libert C (2008). Mx1 causes resistance against influenza A viruses in the Mus spretus-derived inbred mouse strain SPRET/Ei. *Cytokine*.

[79] Nakajima E, Morozumi T, Tsukamoto K, Watanabe T, Plastow G, Mitsuhashi T (2007). A naturally occurring variant of porcine Mx1 associated with increased susceptibility to influenza virus in vitro. *Biochemical Genetics*.

[80] Hijikata M, Mishiro S, Miyamoto C, Furuichi Y, Hashimoto M, Ohta Y (2001). Genetic polymorphism of the MxA gene promoter and interferon responsiveness of hepatitis C patients: revisited by analyzing two SNP sites (-123 and -88) in vivo and in vitro. *Intervirology*.

[81] Pavlovic J, Haller O, Staeheli P (1992). Human and mouse Mx proteins inhibit different steps of the influenza virus multiplication cycle. *Journal of Virology*.

[82] Horisberger MA (1995). Interferons, Mx genes, and resistance to influenza virus. *American Journal of Respiratory and Critical Care Medicine*.

[83] de Jong MD, Simmons CP, Thanh TT (2006). Fatal outcome of human influenza A (H5N1) is associated with high viral load and hypercytokinemia. *Nature Medicine*.

[84] Chan MCW, Cheung CY, Chui WH (2005). Proinflammatory cytokine responses induced by influenza A (H5N1) viruses in primary human alveolar and bronchial epithelial cells. *Respiratory Research*.

[85] Lam W, Yeung AC, Chu IM, Chan PKS (2010). Profiles of cytokine and chemokine gene expression in human pulmonary epithelial cells induced by human and avian influenza viruses. *Virology Journal*.

[86] Lee SMY, Gardy JL, Cheung CY (2009). Systems-level comparison of host-responses elicited by avian H5N1 and seasonal H1N1 influenza viruses in primary human macrophages. *PLoS ONE*.

[87] Perrone LA, Plowden JK, García-Sastre A, Katz JM, Tumpey TM (2008). H5N1 and 1918 pandemic influenza virus infection results in early and excessive infiltration of macrophages and neutrophils in the lungs of mice. *PLoS Pathogens*.

[88] Cameron CM, Cameron MJ, Bermejo-Martin JF (2008). Gene expression analysis of host innate immune responses during lethal H5N1 infection in ferrets. *Journal of Virology*.

[89] Rovin BH, Lu L, Saxena R (1999). A novel polymorphism in the MCP-1 gene regulatory region that influences MCP-1 expression. *Biochemical and Biophysical Research Communications*.

[90] Deng G, Zhou G, Zhang R (2008). Regulatory polymorphisms in the promoter of CXCL10 gene and disease progression in male hepatitis B virus carriers. *Gastroenterology*.

[91] Brull DJ, Montgomery HE, Sanders J (2001). Interleukin-6 gene -174g > C and -572g > C promoter polymorphisms are strong predictors of plasma interleukin-6 levels after coronary artery bypass surgery. *Arteriosclerosis, Thrombosis, and Vascular Biology*.

[92] Zhou J, Law HKW, Cheung CY, Ng IHY, Peiris JSM, Lau YL (2006). Differential expression of chemokines and their receptors in adult and neonatal macrophages infected with human or avian influenza viruses. *Journal of Infectious Diseases*.

